# Trees vs neural networks for enhancing tau lepton real-time selection in proton-proton collisions

**DOI:** 10.1038/s41598-025-04767-x

**Published:** 2025-07-01

**Authors:** Maayan Yaari, Uriel Barron, Luis Pascual Domínguez, Boping Chen, Liron Barak, Erez Etzion, Raja Giryes

**Affiliations:** 1 School of Physics and Astronomy, Tel Aviv University, Ramat Aviv, 69978 Israel; 2 School of Electrical Engineering, Tel Aviv University, Ramat Aviv, 69978 Israel; 3 Department of Physics, University of Toronto, Toronto, ON M5S 1A2 Canada

**Keywords:** Tau lepton, Trigger, LHC, Deep Sets, Experimental particle physics, Scientific data

## Abstract

This paper introduces supervised learning techniques for real-time selection (triggering) of hadronically decaying tau leptons in proton-proton colliders. By implementing traditional machine learning decision trees and advanced deep learning models, such as Multi-Layer Perceptron or residual neural networks, visible improvements in performance compared to standard rule-based tau triggers are observed. We show how such an implementation may lower selection energy thresholds, thus increasing the sensitivity of searches for new phenomena in proton-proton collisions classified by low-energy tau leptons. Moreover, we analyze when it is better to use neural networks vs decision trees for tau triggers with conclusions relevant to other problems in physics.

## Introduction

High-energy proton collision experiments currently face the challenge of recording massive volumes of data at an extremely high rate. The Large Hadron Collider (LHC), a particle accelerator that collides proton bunches at a 40 MHz frequency, exemplifies this issue. The energy deposits and trajectories of particles from proton-proton (*pp*) collisions are recorded by experimental apparatuses, such as the ATLAS and CMS experiments^1,2^. These processes result in a difficult data load to manage within stringent timing constraints. In addition to these technical challenges, while most events contain well-understood physical phenomena that are less critical to study, a minuscule portion of the *pp* collisions unveils rare physics essential for storage and in-depth analysis. Therefore, given the importance of not overlooking these rare events, an extremely efficient filtering mechanism, known as the “trigger system,” is employed. With limited real-time information, the trigger system promptly decides which events warrant storage and further analysis, whereas the remainder are discarded^3^.

The trigger system for ATLAS and CMS consists of multiple stages. The first level (L1) of a multi-level trigger system, which is generally present in hadron collider experiments^4,5^, uses stringent constraints to select the events that their data are kept for the following trigger steps. The L1 trigger is ordinarily implemented on dedicated electronic circuits to speed up the process, while recent implementations use field-programmable gate arrays (FPGAs), allowing a given algorithm to respond promptly. Whether or not an event passes the trigger depends on signals recorded in the different detector subsystems.

Several trigger paths (chains) run in parallel, each with a predefined rate and bandwidth. A key feature in many chains is the energy deposited in the calorimeter. Until recently, simple threshold-based algorithms have been utilized to detect significant energy deposits within the calorimeters, supplemented by isolation criteria to mitigate background rates. Events passing the L1 trigger are further filtered by the high-level trigger (HLT) based on CPUs and GPUs, where more advanced algorithms can be used. However, there have been recent developments in engineering that allow for the implementation of machine learning (ML) methods on FPGAs within the timing and resource constraints necessary at L1^6–9^. These advancements represent a paradigm shift, empowering L1 to execute advanced algorithms previously exclusive to the HLT, thereby enhancing event selection efficiency and broadening the scope of physics analyses within the experiments.

**Fig. 1:**
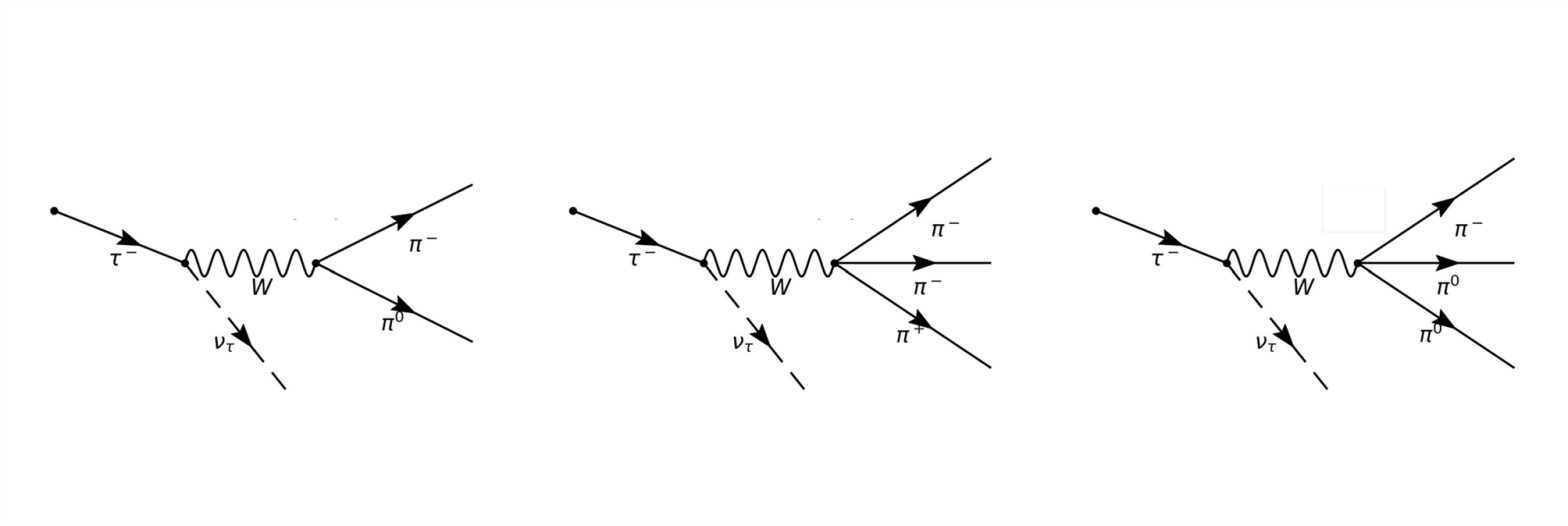
Schematic diagrams of the most frequent hadronic decays of the $\tau$ lepton. The probability of a $\tau$ to decay into hadronic states is 64.79%. Out of which (from left to right) 40% are $\tau \rightarrow \pi ^-\pi ^0$ decays dominated by the $\rho (770)$ meson, 14.4% decay into $\pi ^-\pi ^-\pi ^+$ and 14.3% are $\tau \rightarrow \pi ^-2\pi ^0$ decays^12,13^, both dominated by a $\tau$ decay into $a_1(1260)$ resonance which further decays into $\rho (770) \pi$ intermediate state.

When searching for new physics phenomena, theoretically motivated processes that fall within the abovementioned category include, for instance, those involving hypothesized light particles from so-called hidden sectors. These particles may have couplings to the Standard Model (SM) fields proportional to their mass^10,11^. Consequently, *pp* collision events carrying third-generation fermions, such as the b-quarks or the tau ($\tau$) leptons, are attractive to analyze since they could be sensitive to physics Beyond the Standard Model (BSM) scenarios. However, despite the theoretical solid motivation for storing and analyzing events involving tau leptons, distinguishing between them and most hadronic jet events in a *pp* collider poses a significant challenge. This difficulty primarily arises from the tendency of most tau leptons to decay into hadronic final states (see Fig. 1).

**Fig. 2:**
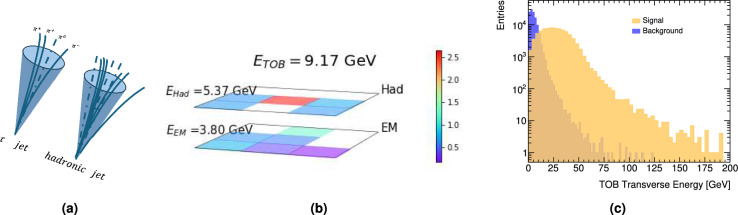
(**a**) A schematic view of a typical tau decay (left) and hadronic jet (right). (**b**) Layer structure of a reconstructed $3\times 3$ TOB matched to the visible component of a hadronically decaying tau lepton. The *x* and *y* axis are the $\Delta \eta$ and $\Delta \phi$ directions. The vertical scale denotes the energy deposited per cell in GeV, also shown per electromagnetic (EM) and hadronic (Had) layers and for the total TOB. (**c**) Transverse energy of reconstructed TOBs for signal and background samples. All reconstructed TOBs are shown for the background, while only TOBs matched to the visible component of a hadronically decaying taus are shown for the signal.

The similarity between the tau hadronic decays and the overwhelming background from jets produced in the hadronization of quarks and gluons characterizing *pp* collisions significantly complicates the selection for tau events (Fig. 2a). This challenge is notably acute in the low-energy regime, where the signatures of the two phenomena are almost indistinguishable, as can be seen in^3^ were the tau trigger efficiency for hadronically decaying tau leptons with transverse momentum from 40 to 25 GeV drastically drops by 30%. Although identification algorithms that leverage advanced deep learning techniques have been developed to distinguish between these particular signatures, as reported in^14^, they are unsuitable for application during the initial stages of the trigger process. This is due to the stringent latency constraints and the limited information available at these early stages.

The adoption of FPGA technology in upgrading the tau trigger promises to enhance algorithmic complexity and effectiveness beyond the capabilities of currently used methods.

This paper explores various machine learning algorithms, highlighting their merits and limitations while considering the trigger system’s hardware and strict latency constraints. The ATLAS collaboration has scheduled the tau trigger system upgrade in two phases: Phase 1 (2019–2022) and Phase 2 (2027–2029). Our discussion sets the stage for potential breakthroughs expected with the Phase 2 upgrade. A notable enhancement in the tau trigger system during the Phase 2 upgrade is the introduction of finer detector granularity. To mimic ATLAS’s data environment, we generate synthetic data that emulates different levels of detector granularity. This enables us to test basic machine learning algorithms on varied granular structures to determine how detector granularity affects algorithm choice. The uncertainty regarding the definitive selection of hardware for the trigger system leads us to evaluate the real-time applicability of each algorithm. We conduct this assessment through a comparative analysis focused on algorithmic parameters indicative of memory consumption. The presented algorithms are tailored for 3D data outputs from the experiment’s calorimeter system, inspired by computer vision techniques applicable to both 2D and 3D image analysis tasks.

This paper is organized as follows. It starts with providing a comprehensive overview of the experimental aspects of triggering tau leptons in *pp* colliders. It sets the stage for introducing the dataset used in the Neural Network (NN) training. Then, it presents the performance outcomes of the various NN architectures studied. Finally, it is concluded by summarising the essential findings and discussing future directions for implementing NN algorithms in fast data processing. The complete implementation of this work can be accessed at^15^.

## Samples and experimental context

This section established the experimental framework essential for comprehending the findings presented in this paper. It comprises two main parts: firstly, an explanation of the detection process for hadronically decaying tau leptons within the ATLAS experiment, including a presentation of the dedicated ATLAS L1 trigger explicitly designed for these events. Secondly, a comprehensive description of our analysis’s signal and background samples offers detailed insights into the experimental methodology.

### Tau leptons in ATLAS

Events with tau particles, the heaviest known leptons, are crucial for understanding the Higgs mechanism and searching for new physics beyond the Standard Model at the LHC. Hadronically decaying tau leptons account for 64.79% of their decay branching^13^, making this channel critical for many exciting processes, such as the $H\rightarrow \tau \tau$ decay. All tau decays occur through the weak interaction, resulting in a tau neutrino that remains undetected due to its minimal interaction. The hadronic products of these events are nearly fully contained within the calorimetric system^16^. ATLAS adopts a right-handed coordinate system centered at the interaction point within the detector. The x-axis extends towards the LHC center, the z-axis runs along the beam pipe, and the y-axis points upwards orthogonally. The polar angle $\theta$ is equivalently expressed as the pseudorapidity $\eta = - \text {ln } \text {tan} (\theta /2)$. The x and y axes form the transverse plane, while the azimuthal angle between them is denoted as $\phi$. The calorimeter system is located around the *pp* collision vertex and measures the energy particles deposited while traversing the material. The calorimeter system measures up to $|\eta |$=4.9 and consists of the electromagnetic calorimeter (ECAL) and the hadronic calorimeter (HCAL). The ECAL absorbs the energy of electromagnetic particles such as electrons and photons. At the same time, the HCAL captures the energy of particles involved in hadronic interactions, including hadronic jets, which are the main background in this study. Each calorimeter is longitudinally divided into three layers with different granularities to help identify and reconstruct incident particles. An additional layer, denominated Presampler or Layer 0, is present in front of the ECAL to correct for energy losses.

Hadronic jets are the most frequent final state in *pp* collisions. They represent a challenging background for this work since their energy deposits in the calorimeter mimic the signature of the final state particles produced in a hadronically decaying tau lepton. No tracking information is available at the L1 trigger. Tracking information could simplify the distinction as most tau jets contain only one or three charged particle tracks, while hadronic jets typically comprise a larger number of tracks.

The L1 tau trigger in ATLAS has evolved significantly over time to adapt to increasingly challenging data-taking conditions and incorporate advancements in firmware development^4,17^. Referred to as L1Calo, this trigger relies solely on information from the calorimeter system. Synchronized with *pp* collisions, L1Calo scans all calorimeter channels to pinpoint regions exhibiting substantial energy deposition. The progression of the L1Calo trigger system and its algorithms from the data-taking period spanning 2015 to 2018 (Run 2) to the current phase (Run 3) is detailed below.

The current L1Calo algorithm to identify tau leptons has a latency constraint of 60 ns, corresponding to 12 clock cycles at 200 MHz^18^. To meet the stringent latency constraints of the L1Calo trigger, calorimeter cells are merged into broader trigger towers spanning both the ECAL and HCAL. In Run 2, the trigger towers had a granularity of $\Delta \eta$ $\times$ $\Delta \phi$ = $0.1\times 0.1$^16^ and longitudinally divided per calorimeter. However, in Run 3, a more granular trigger system was available thanks to upgraded trigger processors^19^, reaching a granularity increase in the $\eta$ direction of up to a factor of 4 in the middle layers of the ECAL^20^.

The search for the most energetic trigger towers is based on constructing 3-dimensional structures known as Trigger Objects (TOBs) within ATLAS calorimeters. Each TOB comprises an $N\times N$ array of trigger towers spanning all layers of the calorimeters, primarily distinguished by its position and energy content. During Run 2, TOBs were characterized by an area of $\Delta \eta \times \Delta \phi = 0.2 \times 0.2$ ($2\times 2$ trigger towers). The selection of TOBs potentially arising from tau candidates relied on a lower transverse energy ($E_{T}$) threshold, calculated as the energy in the transverse plane, coupled with an isolation requirement covering a broader ring, $4\times 4$, surrounding the TOB. This isolation condition aimed at discarding candidates with significant energy in the outer ring, characteristic of jets rather than hadronically decaying tau leptons.

Despite enhanced granularity in Run 3, the approach of identifying and selecting tau TOB candidates remained broadly similar. Run 3 TOBs now encompass cell regions spanning $\Delta \eta \times \Delta \phi = 0.3 \times 0.3$, with varying granularities in Layers 1 and 2. All trigger towers within this region contribute to the characterization of the signal. In Run 3, the isolation requirement is separated from the L1Calo step and conducted in a subsequent L1 trigger stage denominated L1Topo. This separation helps manage the number of selected TOBs by L1Calo, maintaining a manageable rate forwarded to L1Topo. Consequently, in the studies discussed here, the objective is to enhance the rejection capabilities before the isolation stage, leveraging the improved granularity of trigger towers to mitigate the rate burden before isolation imposition.

### Simulated samples

The task of an L1 tau trigger is to classify *pp* collision events into those containing hadronically decaying taus (‘signal’) or those that do not (‘background’). Events classified as signals are subject to further filtering at the HLT.

Simulated samples are used in this study to mimic the calorimeter structure and replicate the triggering strategy employed by ATLAS on hadronically decaying tau leptons. Both signal and background samples are simulated from *pp* collisions at a center-of-mass energy of 13 TeV with MadGraph5_aMC@NLO (version 2.9.5)^21^ for the matrix element computation and interfaced with Pythia8^22^ for the decays, hadronization, and underlying event processes. The signal sample comprises events with hadronically decaying tau leptons obtained from simulated Z boson decays, while the background consists of a sample of events with hadronic jet pairs (dijets).

After the event generation, the smearing of the signals in the calorimeter is performed by Delphes (version 3.4.2)^23,24^. While Geant4^25^ is a detailed simulation environment that provides comprehensive modeling of particle interactions in matter, Delphes is a lighter and faster simulation framework utilizing simplified detector responses suitable for studies of analysis strategies. Delphes introduces smearing terms in the detector resolution and other experimental effects, such as pileup. Pileup refers to additional *pp* collisions simultaneous to the collision of interest. The pileup and the interaction between the particles and the material of the calorimeters are modeled with the ATLAS datacard included in the Delphes package^26^.

To prepare the input dataset, we simulated the TOB reconstruction algorithm used in ATLAS with the granularity of the ATLAS Delphes datacard, which includes a simpler version of the ATLAS calorimeter, in which only an electromagnetic and hadronic layers are present but with flexible granularity as the trigger towers used in the ATLAS L1Calo. Thus, the TOB used in the following consists of a $d\times d$ structure of cells spanning through the two layers of the calorimeter, where *d* takes values of 3, 5, and 9. The motivation for this approach is to evaluate the different architectural performances when increasing the complexity of data. An example of a $2\times 3\times 3$ TOB is illustrated in Fig. 2b, where its total and per-layer energies are also shown.

The signal dataset is specifically prepared to include TOBs originating from tau leptons’ hadronic decay. To achieve this, only the TOBs closest to the truth visible components of the tau lepton decays are considered, with a maximum of two TOBs per event. On the other hand, the background dataset consists of all TOBs reconstructed in the calorimeter, as a single TOB passing the selection criteria will activate the trigger and be recorded. In this work, the complete dataset comprises two types of events: background and signal. Each ‘event’ consists of a batch of TOBs, where the batch size may vary between events. Figure 2c depicts the energy distributions for both signal and background TOBs.

## Supervised learning training

The classification task addressed in this work belongs to the supervised learning category. The model is trained to learn a function that maps inputs to outputs using a labeled training dataset. In the following, a single TOB is called a ‘sample’, representing the individual input provided to all classification models tested in this paper. The problem is approached as a binary classification scenario with a traditional loss function - the cross-entropy metric. We employ the (iterative) Stochastic Gradient Descent algorithm across all the tested models to optimize the cross-entropy loss function. Regarding data balance, the ratio of signal to background TOBs was maintained consistently throughout our discussions, with approximately 70k signal TOBs and 200k background TOBs.

We explored three different learning methods: one is a traditional machine learning model, namely a decision tree, while the other two are NN models. Note that while the model inputs are quite similar to those of ‘standard’ NN tasks, in our case, strict latency and computational constraints arise from the ATLAS experiment’s very high rate of events. Therefore, the goal is to build architectures that are as simple as possible and can still be implemented within FPGAs.

**Fig. 3:**
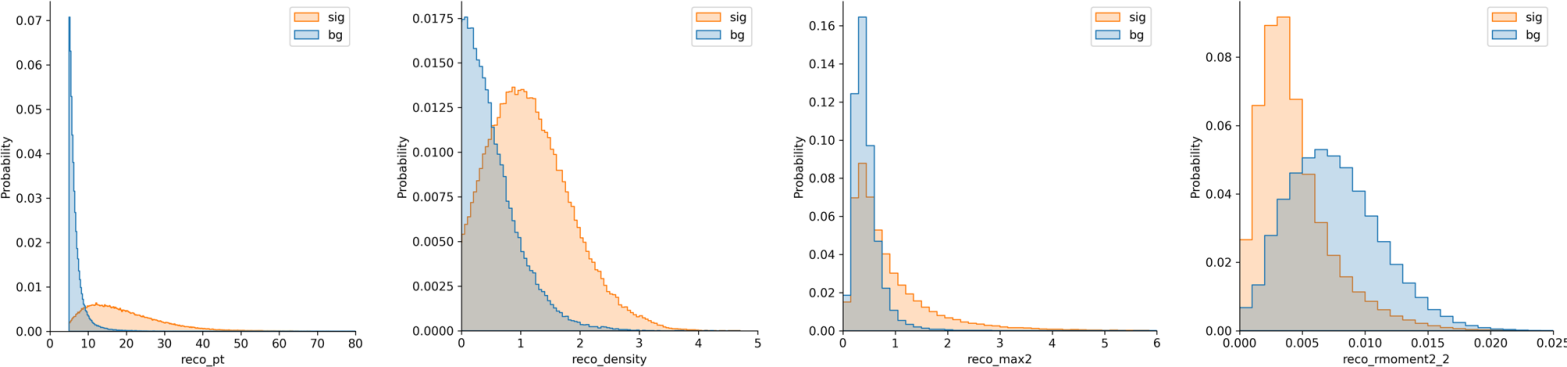
Distributions of a few of the high-level features used to train the XGBoost. Displayed features from left to right: total energy deposited, density of energy concentration in the cells, second largest energy deposit in the second layer, and the second moment of the energy distribution of the second layer.

The first concept we explored is the Extreme Gradient Boosting (XGBoost) algorithm ^27^. XGBoost is a gradient boosting tree model that usually involves a small number of parameters compared to NN models since its inputs are only high-level features produced from the raw data, hence allowing a more straightforward FPGA implementation with minimal computational requirements and low latency inference time^28^. The high-level features we used for this model describe the most important information about the energy deposits and their positions in the TOB in a single vector of size 25. Some of these features are shown in Fig. 3. The complete list of the features, as well as their importance, is listed in the appendix in Fig. 6. These features were calculated independently for each of the three granularities tested. We compared XGBoost to other methods when applied to the high-level features and found that it performed best.

The second architecture is a multi-layer perceptron (MLP) NN, which can learn linear and non-linear relationships between data elements due to the neuron’s activation function. Due to simplicity reasons, we used for the MLP a minimal structure of two hidden layers with (5,4) units each. Unlike in XGBoost, the NN employs all raw data elements in a flattened shape, not as the raw data structure tensor. All energy evidence is transferred as input in this model, but the model does not get explicit spatial information regarding the energy deposit positions in the TOB.

The third approach we study relies on the shape of each TOB and, therefore, treats it as a tensor—similar to a dual channel matrix, where the pixel values are the energy deposits of the cells. For this ‘image-like’ perspective, we built an architecture based on the Residual Network (ResNet)^29^, which consists of skip-connections and contains nonlinearities (using the ReLU activation function as in MLP) and batch normalization layers in between. Specifically, the ResNet was designed as follows: first, a ‘stem’ layer composed of 16 filters with a 3x3 kernel applied with stride 2, batch normalization, ReLU activation, and then MaxPooling. This is followed by two standard ResNet blocks with depth eight. Finally, this is fed into a dense, fully connected layer with a softmax activation. As in the NN, the purpose of this architecture is to transfer energy evidence; however, the physical positions of each cell are also taken into account.

## Results

**Fig. 4:**
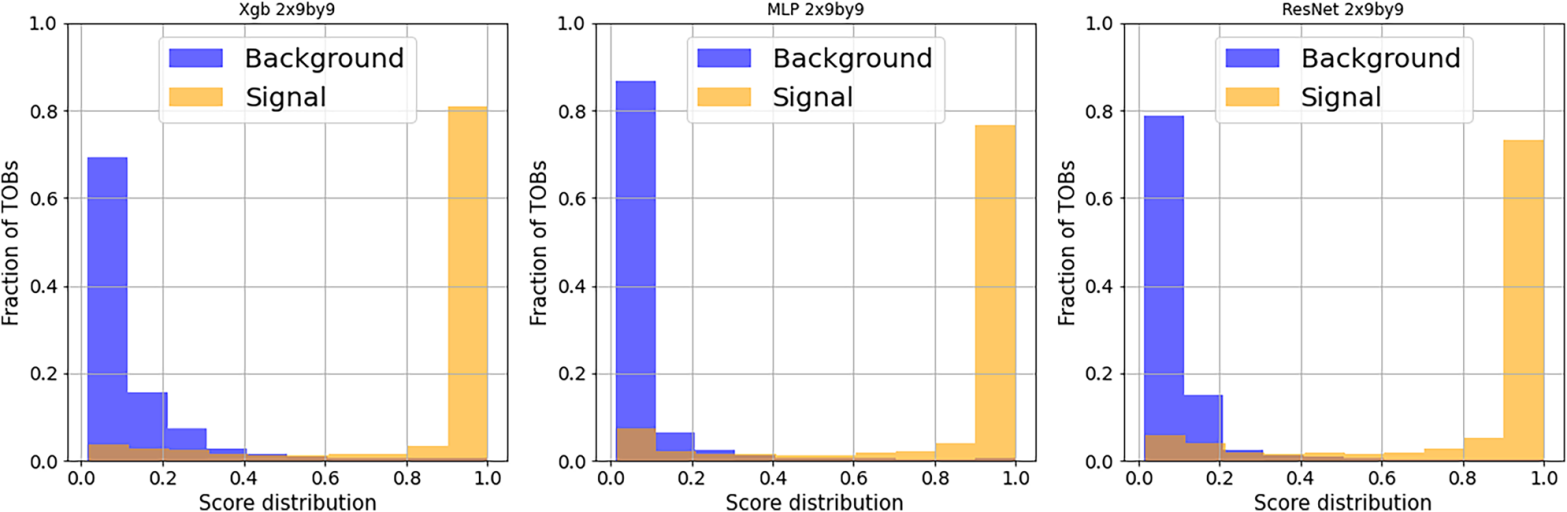
Trigger Objects (TOBs): score distributions for signal (orange) and background (blue) with $2\times 9\times 9$ dimensions for: left: XGBoost; center: MLP; and right: ResNet.

In this section, we discuss the results of the architectures discussed above using the three datasets as mentioned earlier. All datasets include 200k background and around 70k signal samples (TOBs) but with one distinction—the dataset TOBs have a different granularity in the hadronic and electromagnetic layers. Hence, we get three tensor dimensions (this is two layers (EM, HAD), then the size of the towers): $2\times 3\times 3$, $2\times 5\times 5$ and $2\times 9\times 9$. Before evaluating each classifier’s performance, we present the models’ score distribution. In Fig. 4, we demonstrate a good separation between signal and background distributions for all the architectures. However, there is a significant difference between XGBoost and NNs in the variance of the distributions for the high dimensional data structure; the XGB background score variance is higher than NNs’ variance, a feature that might suggest a worse signal/background separation and, therefore a lower classification quality for this kind of data.

The performance is evaluated using multiple metrics; first, we use the most common classification evaluation metrics, namely, the precision-recall curve (PR) and the receiver operating characteristic curve (ROC), which show the performance of a classification model at all classification thresholds by presenting the precision against the recall and true positive rate (TPR) against the false positive rate (FPR) respectively for each. For both curves, we also measure the area under the curve (AUC), an aggregated measurement of the prediction quality across all possible thresholds, with higher values corresponding to better classifiers.

In addition to the previous metrics, we tested a more practical and unique metric, which is more adjusted to the needs of the hadron collider experiments such as the ATLAS experiment: the Turn-on Curve (TOC), also known as an efficiency plot, and its AUC, the TOC-AUC. The TOC shows the efficiency as a function of a given observable obtained from a given model at the **event** level, as opposed to both the ROC and PR curves that quantify it at the TOB level. An event, composed of many TOBs, is classified as a signal if even a single one of its TOBs is classified by the model as a signal. Otherwise, it is classified as background. First, a model threshold is determined by the ‘fake rate’—the maximum number of background events that can be allowed to be mistakenly classified as a signal. The fake rate is dictated by the maximum trigger bandwidth, i.e., the maximal rate of events that can be recorded. Then, given this model threshold, the model efficiency for the signal can be plotted separately for each $\mathrm{p_T}$ range. Since it becomes easier to classify signal events as the tau energy increases, the TOC efficiency is expected to begin at 0 and approach 1. The quicker the ‘turn-on’ response, the more successful the model. The improvement can be seen in the low $\mathrm{p_T}$ ranges of 10–20 GeV. This means with this method, we can detect more low-$\mathrm{p_T}$ taus that would otherwise be missed.

**Fig. 5:**
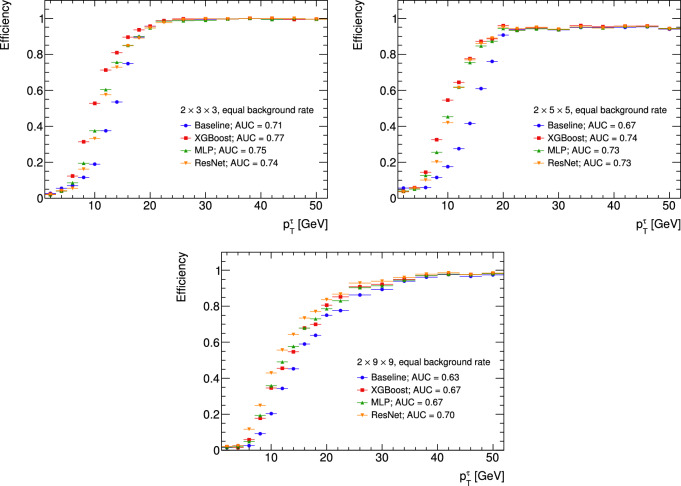
Turn on curve (TOC) of the various architectures with different data dimensions, with area under curve (AUC) shown for each method. In all cases, the ML algorithms significantly outperform the baseline.

**Fig. 6:**
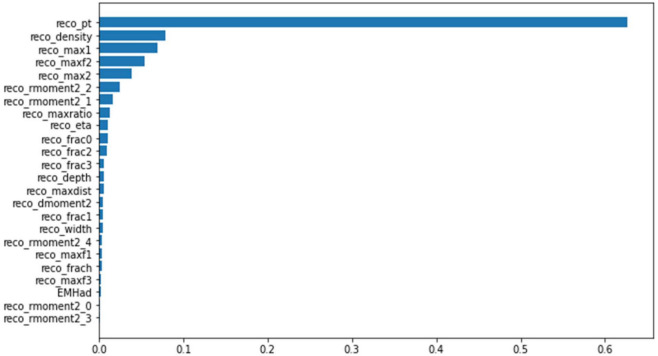
XGBoost Feature importance in descending order.

As seen in Table 1, the regular classification metrics reveal only tiny differences in performance between the three architectures in each data structure. It does not describe the performance vs $\mathrm{p_T}$ and is also affected by the high imbalance of our dataset. This is in contrast to Fig. 5 that presents the TOC curves, where we can evaluate the efficiency of every method: note that the baseline of our task is a traditional algorithm and not an ML one; this algorithm looks for clusters in the TOB layers and above a certain threshold classifies the event as a signal. As we can see from the TOC, all ML algorithms have much higher efficiency for $\mathrm{p_T}$ below 20 GeV and are equal to the baseline performance above this range. The best algorithm in the low $\mathrm{p_T}$ range is changing depending on the data structure we study: in the lowest TOB cardinality $2\times 3\times 3$, the XGBoost performance (in TOC terms for $\mathrm{p_T}$ below 20 GeV) surpasses the NN models. For a slightly higher granularity of $2\times 5\times 5$, XGBoost still takes the lead. However, the performance gap is reduced. Finally, when we run all models over the most extensive data structure $2\times 9\times 9$, the algorithm ranking changes completely, with ResNet performance at the top and XGBoost moving to the bottom. As shown in Fig. 5, the performance of all ML algorithms is superior to the original baseline. It’s also worth noting that we tried training the MLP on the high-level features of the XGBoost, but since these results were consistently strictly worse, this effort was abandoned.

**Table 1:** ROC-AUC, PR-AUC, and F1-MAX metrics, as well as the number of parameters for each of the architectures and per data dimension, are shown.

Metric	Granularity	XGBoost	MLP	ResNet
ROC_AUC	3$\times$3	**0.969**	0.967	0.968
	5$\times$5	**0.936**	0.93	0.931
	9$\times$9	**0.967**	0.963	0.964
PR_AUC	3$\times$3	**0.956**	0.952	0.952
	5$\times$5	**0.912**	0.903	0.904
	9$\times$9	**0.955**	0.95	0.952
F1_MAX	3$\times$3	**0.898**	0.893	0.893
	5$\times$5	**0.846**	0.836	0.838
	9$\times$9	**0.904**	0.9	0.902
# parameters	3$\times$3	1400	97	3778
	5$\times$5	1700	225	10466
	9$\times$9	1900	673	38050

The metrics shown in bold denote which is the best architecture for each of the data dimensions.

To provide some insight into the anticipated memory consumption of each architecture, we examine the count of trainable parameters. As illustrated in Table, the size of XGBoost doesn’t exhibit a significant increase with varying granularity sizes, as the number of input features remains constant across granularities. However, this trend doesn’t apply to the Deep Neural Network (DNN). For instance, the MLP size experiences an impact primarily in the initial layer, which scales quadratically with the granularity dimension and is then multiplied by the number of TOB layers ($L \times d \times d$). In the case of ResNet, the influence of the granularity dimension is even more pronounced, affecting the first layer and subsequent layers. While numerous unexplored parameters remain in determining the optimal algorithm for a given hardware, XGBoost and MLP algorithms present themselves as promising baselines. These algorithms offer the advantage of adjustability based on specific hardware constraints. Subsequently, through a meticulous fine-tuning process, it becomes feasible to evaluate whether the heightened complexity of DNNs is warranted from an efficiency perspective. This evaluation can also show the feasibility of implementing such intricate models for exceedingly high granularity scenarios.

In summary of our findings, we assessed two types of metrics: the conventional binary classification metrics, including ROC_AUC, PR_AUC, and F1_MAX, and the TOC_AUC metric, which relies on $\mathrm{p_T}$. While binary classification metrics excel in straightforward evaluation, the TOC_AUC metric stands out in tasks involving low $\mathrm{p_T}$ regime classification. Regarding binary classification metrics, both XGBoost and NN demonstrate comparable performance, with slight variations based on grid size. However, when considering the TOC_AUC metric, it is advantageous for NN applications in larger grid sizes. Interestingly, a trend emerges within this metric: as the grid size increases, the performance of ResNet experiences significant enhancement. This suggests that in scenarios where the TOC_AUC metric is crucial, XGBoost is preferable for smaller grid sizes. At the same time, NN architectures such as ResNet become increasingly favorable as the grid size expands.

The previous comparison is worthless if we don’t confirm that these architectures fit the existing hardware constraints. To this end, the software hls4ml^30^ was used to translate the neural network model into HLS models. Since the hls4ml doesn’t support customized ResNet layers, instead of using the original ResNet model, an alternative CNN model, using only two simple 2D convolution layers and one fully connected layer, with a similar number of trainable parameters was used to estimate the resource and latency for the CNN/ResNet model. The tests used Vitis HLS 2022.1 with a Xilinx Virtex 7 FPGA XC7VX550T. The default factors for the quantization and parallelization in hls4ml were used. The results are shown in Table 2, including the estimation for the latency, digital signal processing (DSP), flip-flops (FFs), and lookup tables (LUTs). The numbers in the brackets in Table 2 for DSP, FFs and LUTS show the total available resource for the FPGA, . For CNN with 9x9 granularity, the model is too large for hls4ml to translate into the HLS model. For CNN with 5x5 granularity, it is using 213% DSP and 362% LUT, which is more than what Virtex 7 FPGA XC7VX550T can provide.

To conclude, the CNN for all granularities exceeds the available resources at hand and thus is currently not a viable option for the tau trigger. The MLP and the XGBoost, though, are well within the hardware constraints and therefore must be considered. However, it must be noted that the MLP architecture is almost on the edge of the latency constraints of 60 ns within which the current tau trigger algorithm runs for the largest granularities. As seen in Fig. 5, when comparing the TOC_AUC metric, the XGBoost equals or outperforms the MLP for all granularities. For an in-depth discussion on the performance-to-resource tradeoff and optimization of the XGBoost hyperparameters, refer to Chapter 7, part 3 of the following work^31^.

**Table 2:** Latency and resource estimation by hls4ml and Conifer.

	Granularity	XGBoost	MLP	CNN
Latency (ns)	3$\times$3	20	60	320
	5$\times$5	20	65	560
	9$\times$9	20	65	N/A
DSP (2880)	3$\times$3	0 (0%)	85 (3.0%)	2516 (87.4%)
	5$\times$5	0 (0%)	179 (6.2%)	6148 (213.5%)
	9$\times$9	0 (0%)	440 (15.3%)	N/A
FFs (692800)	3$\times$3	4166 (0.6%)	2521 (0.4%)	155345 (22.4%)
	5$\times$5	4166 (0.6%)	5517 (0.8%)	427684 (61.7%)
	9$\times$9	4166 (0.6%)	15019 (2.2%)	N/A
LUTs (346400)	3$\times$3	8320 (2.4%)	2896 (0.8%)	233628 (67.4%)
	5$\times$5	8320 (2.4%)	6731 (1.9%)	1255844 (362.6%)
	9$\times$9	8320 (2.4%)	22117 (6.4%)	N/A

The numbers in the brackets for DSP, FFs, and LUTS show the total available resources for the Xilinx Virtex 7 FPGA XC7VX550T. For each model and granularity combination the estimated resources required is shown for each resource type and in brackets the usage percentage of that respective resource.

## Conclusions

The growing rate of *pp* events at the LHC is vital for searching for new phenomena. However, the increasing pace of collected data poses challenges in filtering the relevant events. A novel tau triggering technique is introduced that can significantly improve the ability to trigger on low $\mathrm{p_T}$ hadronically decaying taus. We introduce a decision tree trained with XGBoost and advanced deep learning techniques such as MLP and ResNet and demonstrate their ability at different complexity levels of data structures. In the future, LHC experiments can utilize these techniques to accommodate the anticipated conditions, which will involve higher detector granularity and more intricate data structures. For most of the energy range considered, ResNet is found to be the best-performing technique for high dimensional structure, while for low complexity data, a traditional ML approach like XGBoost gives the best performance and is also within current hardware and latency constraints. We believe that these observations are also relevant to other scientific problems beyond tau triggers and can guide other researchers in the selection of the machine learning tool to be used for their data.

## Supplementary Information

Supplementary Information.

## Data Availability

The complete implementation of this work can be accessed at: https://github.com/ubarron/tau-trigger;

## XGBoost high level features

### Feature definitions

In total, 24 features were chosen utilizing the lateral radial symmetry of the TOB and the narrowness of tau decay products relative to those of QCD jets. These features were defined using the following notation. $E^l_{h,w}$ is the energy of the cell of layer *l* with height index *h* and width index *w*, where *l* ranges from zero to four with the five indices corresponding to the layers: PS, EM1, EM2, EM3, and HAD. With this notation, the TOB energy of a given layer $E^l$ can be written as:

$$\begin{aligned} E^{l}=\sum _{h, w} E_{h, w}^{l} \end{aligned}$$

The energy of an entire TOB can then be expressed as:

$$\begin{aligned} E^{TOB}=\sum _{l=0}^{4} E^{l} \end{aligned}$$

Using this notation, all the high-level features are given in Table  3.

**Table 3:** Full list of all high-level features used to train the XGBoost and their descriptions

Feature	Definition	Description
pt	$E^{TOB}$	The total energy deposited in the TOB
eta	$|\eta ^{TOB}|$	The absolute $\eta$ value of the central Supercell of the TOB
width	$\frac{\sum _{l, h, w}\left( E_{h, w}^{l} \cdot \sqrt{\left( \Delta \eta _{h, w}\right) ^{2}+\left( \Delta \phi _{h, w}\right) ^{2}}\right) }{E^{T O B}}$	It quantifies how focused the TOB’s energy in its center. The normalized $\eta$ and $\phi$ values, $\Delta \eta ^l_{h,w}$ and $\Delta \phi ^l_{h,w}$, are the distance in $\eta$ and $\phi$ units of cell *h*, *w* of layer *l* from the TOB center, defined as: $\Delta \eta ^l_{h,w}=\eta ^l_{h,w}-\eta ^{TOB}$, $\Delta \phi ^l_{h,w}=\phi ^l_{h,w}-\phi ^{TOB}$, where $\eta ^{TOB}$ and $\phi ^{TOB}$ are the $\eta$ and $\phi$ values of the TOB center. Thus, ‘width’ is the cells average energy fractions multiplied by their radial distance from the TOB center
depth	$\frac{\sum E^{l} \cdot D^{l}}{E^{TOB}}$	The average penetration depth of the energy deposits in the TOB along the calorimeter depth axis. $D^l$ is the depth of the center of layer *l* in cm when measured with respect to the first layer of the calorimeter
density	$\ln \left( \frac{\sum _{l, h, w}\left( \left( 10^{3} \cdot E_{h, w}^{l}\right) ^{2} / V^{l}\right) }{E^{T O B}}\right)$	The ratio of the cell energies squared relative to their respective volumes, where $V^l$ is the volume in ${\hbox {cm}}^3$ of a single cell in layer *l*
frac0	$\frac{E^0}{E^{TOB}}$	The fraction of PS energy out of total TOB energy
frac1	$\frac{E^1}{E^{TOB}}$	The fraction of EM1 energy out of total TOB energy
frac2	$\frac{E^2}{E^{TOB}}$	The fraction of EM2 energy out of total TOB energy
frac3	$\frac{E^3}{E^{TOB}}$	The fraction of EM3 energy out of total TOB energy
frach	$\frac{E^4}{E^{TOB}}$	The fraction of hadronic layer energy out of total TOB energy
max1	$\mathop {max}_{h \in \{1,2,3\},w \in \{1,\ldots ,12\}}\left\{ E_{h, w}^{2}\right\}$	The energy of the cell with the highest energy value in the second layer
max2	$\mathop {max2}_{h \in \{1,2,3\},w \in \{1,\ldots ,12\}}\left\{ E_{h, w}^{2}\right\}$	The energy of the cell with the highest energy in the second layer, excluding the cell with maximum energy (found in previous feature) and its adjacent cells
maxratio	$\frac{max2\left\{ E_{h, w}^{2}\right\} }{max \left\{ E_{h, w}^{2}\right\} }$	The ratio between the two maxima
maxdist	$\Vert \mathop {argmax}_{h,w}\left\{ E_{h, w}^{2}\right\} -$ ${\mathop {argmax2}_{h,w}\left\{ E_{h, w}^{2}\right\} \Vert }_{L_1}$	The Manhattan distance (or $L_1$ norm) between the two maxima cells. If the indices of the maximum cell in layer two are given by $h_1,w_1$ and of the second maximum by $h_2,w_2$, then maxdist can be simplified as $|h_2-h_1|+|w_2-w_1|$
maxf1	$\frac{max \left\{ E_{h, w}^{2}\right\} }{E^{2}}$	The ratio between the maximum and the total energy of the second layer
maxf2	$\frac{max2 \left\{ E_{h, w}^{2}\right\} }{E^{2}}$	The ratio between the second greatest cell energy and the total energy of the second layer
maxf3	$\frac{max \left\{ E_{h, w}^{2}\right\} +max2 \left\{ E_{h, w}^{2}\right\} }{E^{2}}$	The ratio between the sum of both maxima and the total energy of the second layer
emhad	$\frac{\sum _{l=0}^{3} E^{l}}{E^{4}}$	The energy ratio between the electromagnetic layers and the hadronic layer
had3	$\frac{E^{0}+E^{1}+E^{2}}{E^{3}+E^{4}}$	The ratio between the first three layers and the last two
rmoment2_l	$\sum _{h, w} \frac{E_{h, w}^{l} \cdot \left[ \left( \eta _{h, w}-\eta _{C o M}^{l}\right) ^{2}+\left( \phi _{h, w}-\phi _{C o M}^{l}\right) ^{2}\right] }{E^{l}}$	This defines five separate features, one for each layer *l*. For each layer *l*, this defines the energy-weighted second moment of the radial (i.e., longitudinal) distances from the ‘center of mass’ of the TOB. The center of mass is the energy-weighted average $\eta$ and $\phi$ coordinates of the TOB. The center of mass coordinates are defined: $\eta _{CoM}^{l}=\sum _{h, w} \frac{E_{h, w}^{l} \cdot \eta _{h, w}}{E^{l}}$, and $\phi _{C o M}^{l}=\sum _{h, w} \frac{E_{h, w}^{l} \cdot \phi _{h, w}}{E^{l}}$. Although these $\eta$ and $\phi$ coordinates will still typically point to somewhere in the central Supercell of the TOB, they represent a more accurate center than the middle of the central cell
dmoment2	$\sum _{l} \frac{E^{l} \cdot \left( D^{l}-r_{\text{ depth } }\right) ^{2}}{E^{\text{ TOB } }}$	Similar to the previous feature, this feature gives the second moment of the energy weighted distances along the calorimeter depth (i.e. transverse) axis, where $r_{\text{ depth } }$ is the depth feature

### Feature importance

The feature importance for the features above was calculated (see Fig. 6). The metric used was feature ‘weight‘, i.e., the fraction of times the feature was chosen for a split across all the trained trees.
