# Supplementary material for: Trees vs neural networks for enhancing tau lepton real-time selection in proton-proton collisions
Source: Sci Rep. 2025 Jul 1;15:21832. doi: 10.1038/s41598-025-04767-x (PMC12217760; doi:10.1038/s41598-025-04767-x)
Supplement: Supplementary file 1 — Supplementary Information. [file 41598_2025_4767_MOESM1_ESM.pdf]

# Supplementary Information:

## XGBoost High Level Features

### Feature Definitions

In total, 24 features were chosen to utilize the TOB's lateral radial symmetry and the tau decay products' narrowness relative to those of QCD jets. These features were defined using the following notation. $E^l_{h,w}$ is the energy of the cell of layer $l$ with height index $h$ and width index $w$, where $l$ ranges from zero to four with the five indices corresponding to the layers: PS, EM1, EM2, EM3, and HAD. With this notation, the TOB energy of a given layer $E^l$ can be written as:

$$E^l = \sum_{h,w} E^l_{h,w}$$

The energy of an entire TOB can then be expressed as:

$$E^{TOB} = \sum_{l=0}^{4} E^l$$

All the high-level features are given in Supplementary Table 1 using this notation.

### Feature Importance

The feature importance for the features above was calculated (see Supplementary Fig. 1). The metric used was feature 'weight', i.e., the fraction of times the feature was chosen for a split across all the trained trees.

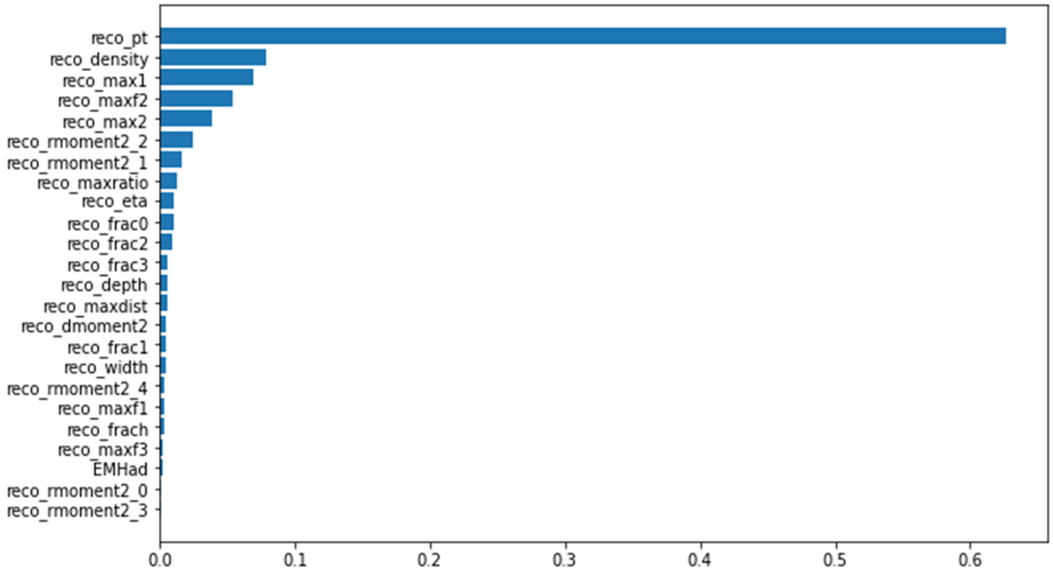


**Supplementary Figure 1.** XGBoost Feature importance in descending order.

**Supplementary Table 1.** Full list of all high-level features used to train the XGBoost and their descriptions.

| Feature | Definition | Description |
|---|---|---|
| **pt** | $E^{TOB}$ | The total energy deposited in the TOB. |
| **eta** | $\|\eta^{TOB}\|$ | The absolute $\eta$ value of the central Supercell of the TOB. |
| **width** | $\frac{\sum_{l,h,w}\left(E^l_{h,w}\cdot\sqrt{\left(\Delta\eta_{h,w}\right)^2+\left(\Delta\phi_{h,w}\right)^2}\right)}{E^{TOB}}$ | It quantifies how focused the TOB's energy in its center. The normalized $\eta$ and $\phi$ values, $\Delta\eta^l_{h,w}$ and $\Delta\phi^l_{h,w}$, are the distance in $\eta$ and $\phi$ units of cell $h,w$ of layer $l$ from the TOB center, defined as:$\Delta\eta^l_{h,w}=\eta^l_{h,w}-\eta^{TOB}$, $\Delta\phi^l_{h,w}=\phi^l_{h,w}-\phi^{TOB}$, where $\eta^{TOB}$ and $\phi^{TOB}$ are the $\eta$ and $\phi$ values of the TOB center. Thus, 'width' is the cell's average energy fractions multiplied by their radial distance from the TOBcenter. |
| **depth** | $\frac{\sum E^l\cdot D^l}{E^{TOB}}$ | The average penetration depth of the energy deposits in the TOB along the calorimeter depth axis. $D^l$ is the depth of the center of layer $l$ in cm when measured with respect to the first layer of the calorimeter. |
| **density** | $\ln\left(\frac{\sum_{l,h,w}\left(\left(10^3\cdot E^l_{h,w}\right)^2/V^l\right)}{E^{TOB}}\right)$ | The ratio of the cell energies squared relative to their respective volumes, where $V^l$ is the volume in $cm^3$ of a single cell in layer $l$. |
| **frac0** | $\frac{E^0}{E^{TOB}}$ | The fraction of PS energy out of total TOB energy. |
| **frac1** | $\frac{E^1}{E^{TOB}}$ | The fraction of EM1 energy out of total TOB energy. |
| **frac2** | $\frac{E^2}{E^{TOB}}$ | The fraction of EM2 energy out of total TOB energy. |
| **frac3** | $\frac{E^3}{E^{TOB}}$ | The fraction of EM3 energy out of total TOB energy. |
| **frach** | $\frac{E^4}{E^{TOB}}$ | The fraction of hadronic layer energy out of total TOB energy. |
| **max1** | $max_{h\in\{1,2,3\},w\in\{1,...,12\}}\left\{E^2_{h,w}\right\}$ | The energy of the cell with the highest energy value in the second layer. |
| **max2** | $max2_{h\in\{1,2,3\},w\in\{1,...,12\}}\left\{E^2_{h,w}\right\}$ | The energy of the cell with the highest energy in the second layer, excluding the cell with maximum energy (found in previous feature) and its adjacent cells. |
| **maxratio** | $\frac{max2\left\{E^2_{h,w}\right\}}{max\left\{E^2_{h,w}\right\}}$ | The ratio between the two maxima. |
| **maxdist** | $\left\| argmax_{h,w}\left\{E^2_{h,w}\right\}-argmax2_{h,w}\left\{E^2_{h,w}\right\}\right\|_{L_1}$ | The Manhattan distance (or $L_1$ norm) between the two maxima cells. If the indices of the maximum cell in layer two are given by $h_1,w_1$ and of the second maximum by $h_2,w_2$, then maxdist can be simplified as $\|h_2-h_1\|+\|w_2-w_1\|$. |
| **maxf1** | $\frac{max\left\{E^2_{h,w}\right\}}{E^2}$ | The ratio between the maximum and the total energy of the second layer. |
| **maxf2** | $\frac{max2\left\{E^2_{h,w}\right\}}{E^2}$ | The ratio between the second greatest cell energy and the total energy of the second layer. |
| **maxf3** | $\frac{max\left\{E^2_{h,w}\right\}+max2\left\{E^2_{h,w}\right\}}{E^2}$ | The ratio between the sum of both maxima and the total energy of the second layer. |
| **emhad** | $\frac{\sum_{l=0}^3 E^l}{E^4}$ | The energy ratio between the electromagnetic layers and the hadronic layer. |
| **had3** | $\frac{E^0+E^1+E^2}{E^3+E^4}$ | The ratio between the first three layers and the last two. |
| **rmoment2_l** | $\sum_{h,w}\frac{E^l_{h,w}\cdot\left[\left(\eta_{h,w}-\eta^l_{CoM}\right)^2+\left(\phi_{h,w}-\phi^l_{CoM}\right)^2\right]}{E^l}$ | This defines five separate features, one for each layer $l$. For each layer $l$, this defines the energy-weighted second moment of the radial (i.e., longitudinal) distances from the 'center of mass' of the TOB. The center of mass is the energy-weighted average $\eta$ and $\phi$ coordinates of the TOB. The center of mass coordinates are defined: $\eta^l_{CoM}=\sum_{h,w}\frac{E^l_{h,w}\cdot\eta_{h,w}}{E^l}$, and $\phi^l_{CoM}=\sum_{h,w}\frac{E^l_{h,w}\cdot\phi_{h,w}}{E^l}$. Although these $\eta$ and $\phi$ coordinates will still typically point to somewhere in the central Supercell of the TOB, they represent a more accurate center than the middle of the central cell. |
| **dmoment2** | $\sum_l\frac{E^l\cdot\left(D^l-r_{\text{depth}}\right)^2}{E^{\text{TOB}}}$ | Similar to the previous feature, this feature gives the second moment of the energy weighted distances along the calorimeter depth (i.e. transverse) axis, where $r_{\text{depth}}$ is the depth feature. |
